# Seroprevalence of brucellosis in communal and smallholder cattle farming in North West Province, South Africa

**DOI:** 10.4102/ojvr.v90i1.2114

**Published:** 2023-12-26

**Authors:** Bontsi Marumo, Tiny M. Hlokwe, Prudence N. Kayoka-Kabongo

**Affiliations:** 1 Onderstepoort Veterinary Research, Agricultural Research Council, Pretoria, South Africa; 2 Department of Agriculture and Animal Health, College of Agriculture and Environmental Sciences, University of South Africa, Pretoria, South Africa

**Keywords:** brucellosis, *B. abortus*, South Africa, cattle, Rose Bengal test, RBT, complement fixation test, CFT, milk ring test, MRT, seroprevalence

## Abstract

Brucellosis is an important bacterial zoonosis responsible for considerable economic losses in livestock and health-related burden worldwide. The objective of this study was to determine the seroprevalence of brucellosis in communal and smallholder cattle farming in four districts of the North West province of South Africa (Dr Ruth Segomotsi Mompati, Ngaka Modiri Molema, Bojanala platinum and Dr Kenneth Kaunda districts). Seven hundred and seventy blood samples from farmed animals (*n* = 378) and abattoir-slaughtered animals (*n* = 392) were collected. In addition, milk samples (*n* = 22) were collected from lactating farmed cows. Rose Bengal test (RBT), complement fixation test (CFT) and milk ring test (MRT) were used to detect antibodies against *Brucella* species. The RBT showed a seroprevalence of 2% at 95% confidence interval (CI: 1.35–3.35), CFT confirmed an overall seroprevalence of 1.95% (95% CI: 1.14–3.12) for all four districts sampled. Although the seroprevalence of brucellosis was found to be low, the possibility of undetected cases of the disease could not be ruled out. Overall, the findings of this study confirmed that brucellosis is endemic in the surveyed areas of the North West province of South Africa.

**Contribution:**

The outcome of this study will contribute to the National Brucellosis Project organised by the Department of Agriculture, Land Reform and Rural Development (2016–2026) to assist in the effective implementation of the disease control measures with a view to prevent further outbreaks in the country’s cattle population.

## Introduction

Brucellosis is a disease caused by the bacterial genus *Brucella* (OIE 2019). *Brucella* is a gram-negative, facultative, and intracellular bacterium that is pathogenic to humans and animals (Bergey & Holt 1993; Madut et al. 2018; Negash & Dubie 2021). *Brucellae* organisms are shed in large numbers in the animal’s urine, milk, placental and other fluids (OIE 2019). A variety of *Brucella* species have been identified, of which four have moderate to significant pathogenicity to humans, and are named from the host source or features of the infection (OIE 2019). These species are *Brucella melitensis* (from sheep & goat), *Brucella suis* (from pigs), *Brucella abortus* (from cattle), and *Brucella canis* (from dogs). *B. melitensis* and *B. suis* have higher pathogenicity to humans while the latter have moderate pathogenicity (OIE 2019). *B. abortus* and *B. melitensis* are the major causes of abortion, birth of weak offspring, stillbirth, retained placenta, and infertility in cows and ewes. Small ruminants brucellosis is mostly caused by *B. melitensis* (World Health Organization [WHO] 2005; Ali et al. 2015). *B. ovis* is also an important cause of orchitis and epididymitis in rams but it is not recognised as a cause of natural infection in goats (WHO 2005). The most effective way of reducing the impact of the disease in livestock and preventing human infection is to control this disease (Pappas et al. 2005).

In South Africa, Brucellosis is a notifiable medical condition in humans (Department of Health 2017; Govindasamy 2020) and a controlled disease in animals (*Animal Diseases Act 35* of 1984). According to the South African legislation (*Animal Diseases Act 35* of 1984 and the *Animal Health Act 7* of 2002), all suspected and confirmed cases of abortion must be reported to the nearest State Veterinary office for zoo-sanitary actions as prescribed in the national bovine brucellosis control scheme. It is also stated in the *Act 35* of 1984 that the responsible person must immunise heifers between the ages of 4 months and 8 months in the Republic of South Africa once with a remedy. The act further emphasises on testing, isolation, branding and slaughtering of infected animals.

Diagnosis of brucellosis is important to monitor the infection for the implementation of effective control measures and epidemiological purposes. Diagnosis of *Brucella* must be carried out on the whole herd because some infected animals show long incubation period and animals may stay serologically negative for a substantial period after infection (OIE 2019). The predicament with brucellosis diagnosis is that one specific method alone is not sufficient to conclude results. Consequently, diagnosis by serology is done using a screening serological test and confirmation test consisting of serology tests or if samples type allow by molecular or other supportive diagnostic tests (Bergey & Holt 1993; Wang et al. 2014). It is generally recommended that the RBT must be used in combination with other standard serological tests for more reliable detection and to avoid false positives (OIE 2019). Another challenge with serological methods is that they cannot differentiate between true infections to vaccine strains such as S19 and RB51 (OIE 2019).

The *South African Animal Diseases Act 35* of 1984 recommends immunisation of heifers between 4 months and 8 months. This is mainly because vaccination with S19 may interfere with serological tests while RB51 does not react with smooth strains in serological tests (OIE 2019). Another challenge with RB51 is that it could cause abortions in pregnant cows and also does not provide lifelong protection like S19 (OIE 2019). In general, a highly effective vaccine has not been developed. Despite the fact that the S19 and RB51 vaccines have been effective in controlling the state of brucellosis in many countries, various challenges have been reported leading to an ongoing research to develop a vaccine without drawbacks (Dorneles, Sriranganathan & Lage 2015).

Among other challenges, the current vaccines have been reported to interfere with the diagnosis of brucellosis in laboratories (Ducrotoy et al. 2017). Due to its zoonotic nature and its negative impact on livestock and human health, research on a vaccine that will address the current challenges is vital (Saeed et al. 2020). In the absence of an effective vaccine, it is difficult to determine the seroprevalence of brucellosis in cattle in the North West (NW) province of South Africa. Availability of an effective vaccine will contribute towards a sustainable strategy for control of this zoonotic disease. Meanwhile, the success of the current vaccines also depends on the cooperation of farmers with the veterinarians, animal health technicians and the laboratories (DAFF 2016c).

Currently, there are very few publications on the seroprevalence of brucellosis in the communal and smallholder farming areas in South Africa including the NW province. The disease is endemic in South Africa (Simpson et al. 2021). The economic implications of brucellosis are a threat to the development of the agricultural sector, particularly in communities practising communal livestock management systems (Lokamar et al. 2020). Its zoonotic nature makes brucellosis a burden to society. In recent years, an increase of brucellosis outbreaks has been reported in the different provinces; hence, the Department of Agriculture, Land Reform and Rural Development (DALRRD) initiated a national brucellosis project to assess the status of brucellosis in the different provinces.

## Materials and methods

### Study area, design and sampling strategy

**FIGURE 1:**
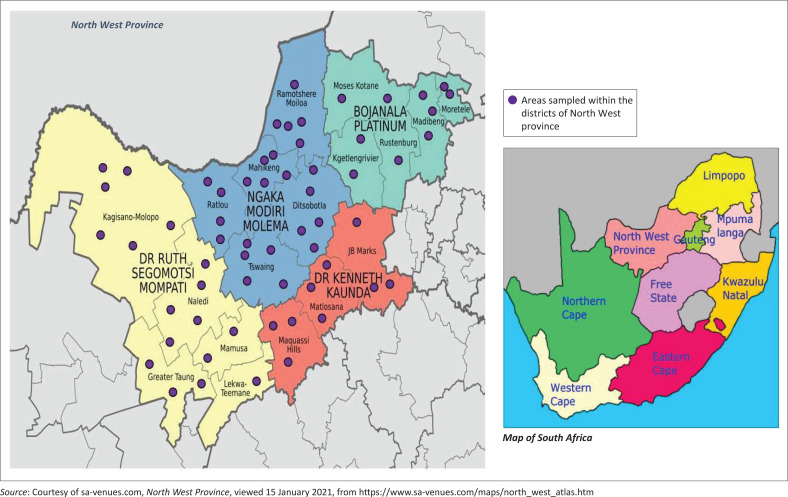
Sample collection site (indicated in purple-coloured marks).

This study was conducted in selected communal, commercial and non-commercial farms of the NW province, and was part of an umbrella project with the goal to produce comprehensive data on the prevalence, distribution, risk factors and zoonotic implications of brucellosis in the study areas. A cross-sectional design with a multistage sampling strategy was used. Samples were collected from abattoirs and farms in all four major districts of the NW province namely, Dr Ruth Segomotso Mompati, Dr Kenneth Kaunda, Bojanala Platinum, and Ngaka Modiri Molema, under the supervision of a veterinarian (Figure 1). The sampling frame included all sub-districts that are more rural in communal production setting in the selected areas. Villages and dip tanks in these municipalities were selected in collaboration with the provincial Department of Agriculture (veterinary services) based on accessibility, livestock population, perceived history of zoonoses such as brucellosis, and collaboration from communities. The animals included in this study were also selected conveniently at the time of visit at each village and/or dip tank and/or abattoir. To avoid duplication and sampling in areas beyond the province, a sample collection sheet was used. This sample sheet included information on the age, sex, farm name and GPS co-ordinates of the location of the farm or abattoir.

### Source of samples and animal population

Consent to sample animals was obtained from participating farmers. The study population included all cattle above the age of 2 years. In all farms, animals that have recently aborted or have the history of abortion were sampled; otherwise, animals were conveniently selected. Blood and milk from lactating cows were collected in the current study. Consent from the managers of abattoirs was obtained prior to the visits. This study targeted both high- and low-throughput abattoirs. This is mainly because high-throughput abattoirs mostly deal with the same farms with which there is a contract agreement, while low throughput is mostly servicing anyone in the rural areas. On the day of the visit, all condemnations data and reason for condemnation were obtained from the meat inspector on duty. Retrospective historical information was also assessed from the previous records. Information regarding the type of livestock, breed, sex, age, the origin and management system (if available) for the abattoir animals were also obtained. A total of 792 samples were collected from farmed (blood: *n* = 378 and milk: *n* = 22) and abattoir animals (blood: *n* = 392).

### Sample size determination and data collection

The number of cattle sampled was determined using the epidemiological formula as described by Thrusfield (2007) and the EPITOOLS software for calculations (Thrusfield 2007). The values used in the calculation included estimated prevalence at 0.5, desired precision at 0.05, confidence level at 0.95 with an estimated population size of 10 000 (Daniel 1999). The total sample size calculated was 385. This resulted in 385 samples to be collected from live farmed animals, and 385 from cattle slaughtered from abattoirs, resulting in a total of 770. However, the required number of samples collected from farms could not be reached; hence, more samples were collected from abattoir animals to achieve the required sample size (Table 1). To avoid duplication of sampled farms, the sample collection form was completed for record purposes. Samples were collected by animal health technicians under the supervision of the state veterinarians before being analysed at Onderstepoort Veterinary Research (OVR).

**TABLE 1:** Number and types of samples collected per district in the North West province.

Type of samples	Name of district and number of samples				Total per sample type
	Ngaka Modiri Molema	Dr. Kenneth Kaunda	Dr Ruth Mompati	Bojanala Platinum	
Blood samples from slaughter animals (abattoirs)	113	93	66	120	**392**
Blood samples from farms	124	103	105	46	**378**
Milk samples from farms	5	3	5	9	**22**

**Total per district**	**242**	**196**	**176**	**175**	**792**

### Types of samples

Approximately, 7.5 mL of blood (*n* = 378) was collected in red top vacutainer tubes from the jugular and coccygeal veins of the live farmed cattle. Tubes were centrifuged at 1500 × *g* for 15 min; sera were decanted into sterile tubes and stored at 4 °C for short-term storage before processing and at –20 °C for long-term storage. The blood from abattoirs was collected during slaughtering using vacutainer tubes and the same procedure as with blood from live animals was followed. Milk samples were collected from individual lactating cows upon the farmer’s consent. Most farmers were, however, reluctant to allow milk sampling. The milk samples (*n* = 22) were collected in sterile screw-capped bottles and transported on ice to OVR institute for analysis.

### Serological methods

Serological procedures that were carried out included the Rose Bengal test (RBT), the complement fixation test (CFT) and the milk ring test (MRT). These methods were validated by the OVR bacteriology serology laboratory using proficiency testing samples (Table 2).

**TABLE 2:** The sensitivity and specificity values obtained from validation are indicated in the table.

Parameters	RBT	CFT	MRT
**Sensitivity (%)**	99.15%	99.03%	100%
95% CI	95.37% – 99.98%	94.71% – 99.98	95.85% – 100.00%
**Specificity (%)**	100.00%	100.00%	100%
95% CI	94.56% – 100.00%	93.94% – 100.00%	87.66% – 100.00%

*Source*: Potts, A., unpublished data: Bacterial serology BS/ME 001,003,005

RBT, Rose Bengal test; CFT, complement fixation test; MRT, milk ring test; 95% CI, 95% confidence interval.

### Rose Bengal Test

The RBT was used to detect anti-*Brucella* antibodies in all 770 sera as per laboratory procedure (OIE 2019). Sera from 378 farm-based and 392 abattoir-slaughtered animals were tested. The test utilised *B. abortus* RBT antigen (Onderstepoort Biological Products, South Africa). A total amount of 25 µL of serum and 25 µL of antigen was dispensed into each well of white porcelain hemagglutination plates. The plate was then allowed to mix and incubated for 4 min on the shaker set at 40 revolutions per minute (rpm). The results were observed on the ultraviolet light box, and positive sera were subjected to the CFT for brucellosis confirmation as previously described (OIE 2019).

### Complement fixation test

The CFT involves inactivation and serial dilutions of sera, reagent dispensing and relevant incubation at different phases. The first stage involved the antigen mixed with the complement, then if the test serum contained antibodies to the antigen, the complement would not get fixed and would not react in the second stage. In the second stage, sheep red blood cells mixed with anti-sheep antibody were added. If the complement has been fixed in the first stage, no haemolysis would take place. This is considered positive as it would mean the serum contained *Brucella* antibodies. Haemolysis of red blood cells indicates that the test is negative as the antigen was not fixed (OIE 2019).

### Milk ring test

Milk (*n* = 22) was subjected to MRT using *B. abortus* MRT antigen (Onderstepoort Biological Products, South Africa). The MRT involves mixing 30 µL of antigen with 1 mL of milk in a non-sterile plastic tube (1 mL – 5 mL) with a screw top. The tubes were then inverted ± 5 times to ensure thorough mixing and incubated at 37 ^o^C ± 2 ^o^C for 1 h before reading the results (OIE 2019). Homogenised, pasteurised or sour milk was not tested as it would interfere with the test results.

### Statistical data analysis

The data obtained were entered into Microsoft Excel^®^ (Microsoft, United States) database and descriptive statistics were generated. The association between different variables and knowledge on farm practices regarding zoonoses was assessed by Chi-square (χ^2^) test. Odds ratios (OR) and confidence intervals (CI: 95%) were calculated to assess potential risk indicators associated with brucellosis seroprevalence in a univariate logistic regression model. An equation for apparent prevalence was used to calculate the percentage of positive animals (% positive) where apparent prevalence was

$$=\frac{\text{Number of positive animals}}{\text{Number of animals tested}}100\%.$$ [Eqn 1]

The true prevalence estimate was calculated using an equation:

$$\text{Var}(\text{AP})=\frac{\text{AP}(1-19)}{\text{n}{\left(\text{se}+\text{sp}\right)}^{2}},$$ [Eqn 2]

adopted from Cameron and Trivedi (2001) which relates to sensitivity and specificity (Cameron & Trivedi 2001). Variance (Var) for the apparent prevalence (AP) was estimated of variance for the apparent prevalence, AP was used for apparent prevalence, Se for sensitivity of CFT test, and Sp specificity, for serology assays. The calculation of the 95% CI for the true prevalence was performed using the following equation:

$$\text{AP}-\left(\text{Z}\alpha \text{ X }\sqrt{\mathrm{var}(\text{AP})}\right);\text{AP}+\left(\text{Z}\alpha \text{ X }\sqrt{\mathrm{var}(\text{AP})}\right),$$ [Eqn 3]

where Zα at a 95% confidence level is 1.96.

### Ethical considerations

Ethical clearance was obtained from the College of Agriculture and Environmental Science (CAES) (Ref. 2020/CAES_AREC/123) and the OVR animal ethics guidelines and regulations (AEC 18.17). Section 20 approval was granted according to *Act 35* of 1984 by the Directorate of Animal Health, South Africa (Ref. 12/11/1/1 [729]). Approval for sampling in abattoir facilities was granted by the NW director of veterinary services (Ref. 12/15/1).

## Results

The district distribution, age and sex of the animals as well as abortion status of the cows from which samples were collected in communal (practiced mostly by rural households), commercial (farming of cattle and other livestock for money), and non-commercial (undertaken to provide for family and not to generate income) farms of the NW province (Table 3).

**TABLE 3:** Summary of distribution of animals sampled and tested for brucellosis in the North West Province according to district distribution, sex, and abortion status.

Variable	Level	Ngaka Modiri Molema	Dr Kenneth Kaunda	Dr Ruth Mompati	Bojanala Platinum	Total
Distribution	Number of samples	237	196	171	166	770
	Percentage	30.78	25.45	22.21	21.56	100
Sex	Male	56	76	51	63	246
	Female	181	120	120	103	524
Abortion history	Abortion	8	10	5	5	28
	No abortion	85	93	74	14	266
Location	Abattoir	113	93	66	120	392
	Farm	124	103	105	46	378

An overall animal participation of 30.78% (237/770) in the Ngaka Modiri Molema district, 25.45% (196/770) from Dr Kenneth Kaunda district, 22.21% (171/770) from Dr Ruth Mompati, and 21.56% (166/770) from Bojanala Platinum districts was achieved. Out of the 770 cattle sampled, milk was collected from only 2.86% (22/770) of the lactating cows.

Overall, the total number of female animals tested were 68.05% (*n* = 524) and 31.94% (*n* = 246) animals were males. There was a significant difference in the sex of animals sampled between districts with more female cattle sampled (*p* = 0.002, *df* = 3, χ^2^ = 14.85). The abortion status could only be determined for 294/524 (56.10%) of the farmed cows, and abortions were reported in 5.34% (28/524) of the cows. The least number of abortion cases were reported in both Dr Ruth Mompati and Bojanala Platinum districts at 1.70% (5/294) each. Dr Kenneth Kaunda district had 3.40% (10/294) of abortion cases, followed by Ngaka Modiri Molema with 2.72% (8/294) cases. The results showed no significant difference in abortion statuses amongst districts (*p* = 0.064, *df* = 3, χ^2^ = 7.25).

A total of eight cattle breeds were sampled in this study. The most samples were collected from Bonsmara at 41.43% (319/770), followed by Nguni at 25.32% (195/770). Other breeds were New Jersey at 13.40% (103/770); Brahman, 7.64% (59/770); Mixed Breed 7.9% (61/770); and Afrikaner, 3.77% (29/770). The least sampled breed was the Holstein Friesian at 0.52% (4/770).

### Rose Bengal test

A screening test using the RBT was performed on all sera (*n* = 770) as per laboratory procedure (OIE 2019). Sera from (*n* = 378) farm-based and (*n* = 392) abattoir-slaughtered animals were tested. Only 2.3% (18/770) of the samples tested positive for antibodies against *B. abortus*, which was indicated by agglutination. Agglutination was observed in sera from 3.17% (12/378) farmed animals and 1.53% (6/392) abattoir-slaughtered animals. The overall seroprevalence for RBT positive was found to be 2.3% at 95% confidence interval (CI: 1.35–3.35).

Individually, the most positive reactors were collected from Ngaka Modiri Molema district at 4.64% (11/237), followed by Dr Ruth Mompati and Dr Kenneth Kaunda at 2.52% (5/171) and 1.02% (2/196) respectively. No positive RBT results were identified from the Bojanala Platinum district. A total of 97.6% (*n* = 752) sera tested negative as no agglutination was observed.

### Complement fixation test

All (*n* = 18) samples recorded as positive for RBT were subjected to the CFT which was used as a confirmatory serological diagnosis test for detecting the presence of *Brucella* antibodies (antibodies against *B. abortus*). The CFT results indicated that out of the 2.3% (18/770) samples that tested positive for RBT, only two (*n* = 2) samples were negative as indicated by complete hemolysis in microtitre wells. The negative results from this confirmatory test were from abattoirs in the Dr Kenneth Kaunda district. This resulted in a total of 2.07% (16/770) samples that were confirmed positive for *Brucella* antibodies by the CFT as indicated by the absence of haemolysis.

Overall, CFT positive results were detected only in Ngaka Modiri Molema (1.42%, *n* = 11/770) and Dr Ruth Mompati (0.64%, *n* = 5/770) districts. The results indicated the overall CFT prevalence of 1.95% (95% CI: 1.14–3.12).

It should be noted that the interpretation of serological results depends on several factors such as infections status, vaccination status (S19 or RB51), incorrect and irresponsible use of the S19 vaccine, current pregnancy status, date of calving or abortion, age of animal, previous titres and possible exposure to infection. In this project, in most of the cases, the vaccination status was unknown as farmers could not confirm the vaccination status of the herd (DAFF 2016a). The DALRRD recommends CFT positive levels to be set from 30 CFT IU/mL for calfhood vaccination or unvaccinated or unknow vaccination status and from 60 CFT IU/mL for adult vaccinated animals. Titres of 18–24 are deemed suspicious for unvaccinated, calfhood vaccination or animals with an unknown history of vaccination (DAFF 2016a).

### Milk ring test

The MRT was conducted on samples collected from lactating cows. Milk samples could only be obtained from 5.82% (*n* = 22/378) cows during convenient sampling. All collected milk samples reacted negatively to the MRT. A lighter shade cream layer was observed on the milk which is an indication of a negative test result.

## Discussion

### Rose Bengal test and complement fixation test

Serological results obtained indicated the overall prevalence of brucellosis to be 2% (95% CI: 1.35–3.35) with the RBT as a screening test. According to previous reports, RBT could demonstrate false-positive results because of non-specific serological reactions that may occur or because of animal vaccination with the S19 strain. It is for this reason that all RBT reactors were confirmed by CFT (DAFF 2016c). Of the 2.07% (*n* = 16/770) samples that tested positive with CFT, three were found to have low titres; however, these results were still within the required titre range to be regarded as positive. According to the DALRRD’s bovine brucellosis manual and other reports, cattle with antibody titre values of ≥ 30 IU/mL are regarded as positive (DAFF 2016b; Godfroid et al. 2004). Of the four districts studied, samples with positive CFT results originated from Ngaka Modiri Molema and Dr Ruth Mompati with seroprevalence of 4.65% (95% CI: 2.61–8.11) and 2.34% (95% CI: 0.91–5.85), respectively. A possible explanation of the Kenneth Kaunda samples that tested RBT positive and negative for CFT could be the presence of IgM because of some cross-reacting antibodies or as a result of vaccination with the S19 strain (Nielsen 2002). Looking at the distribution and number of samples tested, there is a possibility that the other two districts (Dr Kenneth Kaunda and Bojanala Platinum) are not necessarily free of brucellosis as a limited number of animals were sampled compared to the other two districts. This was mainly because of farmers’ lack of cooperation to participation in the study. Only 12.16% (*n* = 46/378) samples could be obtained from the Bojanala Platinum farmers and 23.72% (*n* = 93/392) from abattoirs in Dr Kenneth Kaunda district. Based on the interpretation of the RBT and CFT results, the overall brucellosis seroprevalence was found to be 1.95% (95% CI: 1.14–3.12).

Samples that tested positive in the RBT screening but negative upon CFT confirmation were collected from cattle originating from Dr Kenneth Kaunda district. All confirmed CFT positive reactors from Dr Ruth Mompati were collected from cattle at one abattoir. It should be noted that this abattoir seemed to have challenges regarding cleanliness and hygiene in general. Researchers observed that the abattoir had very limited spaces, with animals coming into contact with one another, and workers wore dirty overalls which seemed to be protective clothing worn the previous day of slaughtering. The possibility of cross-contamination during bleeding and slaughtering of the animals with positive results could not be overlooked as previously reported (Ntirandekura et al. 2018).

The eleven (*n* = 11, 1.43%) positive reactors from Ngaka Modiri Molema districts were collected from two different abattoirs (one positive each), and from four farms (two positives each), and another farm with only one positive cattle. The obtained prevalence in this study was low and in agreement with one research outcome previously conducted in NW province. The study was conducted from 2007 to 2015 and recorded a prevalence of 6.31% in cattle from the NW province (Kolo et al. 2019).

Although Kolo and co-authors included other livestock species such as sheep, goats and pigs (samples submitted over a 9-year period at the OVR institute), cattle brucellosis had the highest occurrence in all nine provinces of South Africa (Kolo et al. 2019). Another retrospective study that was conducted between 2009 and 2013 in the Bojanala district revealed an overall herd prevalence of 33.33% and 3.18% individual prevalence in dairy, commercial and communal cattle (McCrindle, Manoto & Harris 2020). The latter had the lowest individual prevalence considering the extent of the study period. It should also be noted that the study was conducted in only one district of the NW province; hence, the prevalence may have been higher if all districts were sampled. This prevalence is consistent with finding by Modisane and co-authors (Modisane 2019), whereby a 7.7% seroprevalence was obtained over a 7-year study period at Mabeskraal village (Modisane 2019). Outcomes of the current study support the hypothesis that the prevalence distribution may not be different from other areas with similar zoo-epidemiological situations (Kolo et al. 2019).

### Milk Ring test

In addition to RBT and CFT, the MRT was carried out on milk samples obtained from lactating cows. Most farmers were, however, reluctant to allow milk sampling and those who were willing did not have lactating cows, as such, there was a limited number of milk samples collected in this study. All milk samples tested negative to antibodies against *B. abortus* when subjected to MRT. The results of the MRT corroborated the RBT and CFT results obtained from the same (corresponding) animals. The advantage of MRT is that it is inexpensive as milk can be pooled from several cows from one farm (OIE 2019) although milk from individual cows was analysed in the current study. This method, however, has a disadvantage that the milk: antigen ratio in bulk samples often makes it difficult to detect a small number of animals in a large herd (DAFF 2016a). Another challenge with the MRT is that late lactation cycle may produce false reactions for cows that are vaccinated by S19 in less than 4 months before testing (Ducrotoy et al. 2017).

### Limitations of the study

The limitations encountered during this study included the refusal from some farms and abattoirs’ owners to grant the permission to collect samples from their premises because of the fear of coronavirus disease 2019 (COVID-19) infection. In addition, only a limited number of milk samples could be collected because of a belief by farmers that sampling lactating cows will affect milk production and make the calves sick. The funds allocated to this project were available for a defined period and this project was also part of a Masters’ degree programme which was also time bound. The smooth running of this project was disturbed by the COVID-19 pandemic.

## Conclusion

The aim of this study was to determine the seroprevalence of Brucellosis in communal and smallholder cattle farming in the NW province of South Africa. The prevalence of brucellosis was found to be low at 1.95% (95% CI: 1.14–3.12) in the four main districts of the NW province. Although the prevalence was low, the possibility of undetected cases of brucellosis cannot be ruled out in all districts, especially in the Bojanala Platinum and Dr Kenneth Kaunda districts where a limited number of cattle were sampled. The fact that farmers were reluctant to provide milk from their lactating cows could also be the reason for the low prevalence.

Another challenge was that samples were collected in 2020 and 2021 which was during COVID-19 pandemic and neither vaccination against brucellosis nor any other did take place on those farms. There was less movement to no movement of cattle during the pandemic. We also noted uncertainty about history of types of vaccination administered previously in most of the farms. To have a sustainable strategy for controlling brucellosis, the study recommends full enforcement by the South African government for compliance to the legislation which includes vaccination of heifers, test, and slaughter and compulsory testing before selling cattle. Due to its chronic nature, if not controlled, the spread of disease to uninfected cattle herds will continue. Indeed, the effective implementation of brucellosis control as a priority of the South African Veterinary Strategy plan (2016–2026) is crucial.
